# *In-vivo* full-field measurement of microcirculatory blood flow velocity based on intelligent object identification

**DOI:** 10.1117/1.JBO.25.1.016003

**Published:** 2020-01-22

**Authors:** Fei Ye, Songchao Yin, Meirong Li, Yujie Li, Jingang Zhong

**Affiliations:** a Jinan University, Department of Optoelectronic Engineering, Guangzhou, China; b Sun Yat-sen University, Third Affiliated Hospital, Department of Dermatology, Guangzhou, China; c Sun Yat-sen University, Sixth Affiliated Hospital, Reproductive Medicine Center, Guangzhou, China

**Keywords:** biomedical optics, biophotonics, medical imaging

## Abstract

Microcirculation plays a crucial role in delivering oxygen and nutrients to living tissues and in removing metabolic wastes from the human body. Monitoring the velocity of blood flow in microcirculation is essential for assessing various diseases, such as diabetes, cancer, and critical illnesses. Because of the complex morphological pattern of the capillaries, both *in-vivo* capillary identification and blood flow velocity measurement by conventional optical capillaroscopy are challenging. Thus, we focused on developing an *in-vivo* optical microscope for capillary imaging, and we propose an *in-vivo* full-field flow velocity measurement method based on intelligent object identification. The proposed method realizes full-field blood flow velocity measurements in microcirculation by employing a deep neural network to automatically identify and distinguish capillaries from images. In addition, a spatiotemporal diagram analysis is used for flow velocity calculation. *In-vivo* experiments were conducted, and the images and videos of capillaries were collected for analysis. We demonstrated that the proposed method is highly accurate in performing full-field blood flow velocity measurements in microcirculation. Further, because this method is simple and inexpensive, it can be effectively employed in clinics.

## 1 Introduction

Microcirculation, the phenomenon of blood circulation in the smallest blood vessels of diameter 10 to 200  μm,^1^ plays a key role in delivering oxygen and nutrients to living tissues and in removing metabolic wastes. Monitoring the blood flow in microcirculation helps diagnose various diseases, such as diabetes, cancer, and other critical illnesses.^2^^–^^4^ By monitoring the microcirculation of skin, the microvascular changes in the morphology and function of the nailfold area of the finger have been studied.^5^

Laser Doppler imaging (LDI),^6^ laser speckle imaging (LSI),^7^^–^^9^ and optical capillaroscopy (OC)^10^^–^^12^ are the chief techniques currently involved in microcirculation research. However, these methods possess a few limitations. For example, the LDI can obtain the average changes in blood flow in large tissues but cannot measure the blood flow velocity of an individual blood vessel in absolute values. Similarly, the LSI can provide a semiquantitative real-time mapping of flow fields, but it has to be calibrated. Further, the results are in arbitrary units and are not directly related to the actual flow values. Moreover, the conventional OC methods, including the frame difference method and the optical flow method,^13^^,^^14^ can visualize some capillaries under the nailfold and skin, but their blood flow velocity measurements rely on computer video processing. Calculations in the frame difference method are simple and update quickly; however, this method is vulnerable to image noise, and the result heavily depends on the threshold selection in the algorithm. In addition, when the target is stationary for a long time or the amplitude of the motion is small, this method does not perform well. The optical flow method, when employed in detecting moving objects, creates very large amount of data for calculation, and thus, the real-time performance and practicality are severely affected. Moreover, owing to the low signal-to-noise ratio of the microcirculation images and the complex morphological pattern of the capillaries, *in-vivo* full-field flow velocity measurement of capillaries using the conventional OC methods is challenging.

Machine learning and deep learning have been widely used in image processing.^15^^–^^25^ Traditional machine learning has been successfully applied in detecting blood vessels and measuring blood flow velocity in capillaries of nailfold automatically.^21^^–^^23^ Deep learning that uses deep neural network (DNN) is a particular kind of machine learning. Compared with traditional machine learning algorithm, deep learning technology is easier to adapt to different scenarios so it holds a high potential for application in automatic identification and segmentation of blood vessels in medical images.^18^^,^^24^^,^^25^

A spatiotemporal-diagram-based method has been proposed to perform average blood flow measurements,^26^^,^^27^ which has the merits of high measurement accuracy, less influence of noise on the measurement results, and high calculation speeds.

In this research paper, we develop an *in-vivo* optical microscope with a light source of 420 nm. In addition, we propose an *in-vivo* full-field microcirculation velocity measurement (FMVM) method based on intelligent object identification. The FMVM method combines the DNN model (for automatic identification of capillaries) and the spatiotemporal diagram method (for flow velocity measurement), to achieve full-field flow velocity measurement of microcirculation. In this research, the microcirculation images of the face, arm, and other parts of the body were collected to create a dataset for DNN model training. Furthermore, experiments such as intelligent microcirculation identification and segmentation, flow velocity measurement of microcirculation, and *in vivo* FMVMs were conducted. The trained DNN model demonstrated superior performance over the threshold segmentation and frame difference methods, in microcirculation identification and segmentation. Moreover, the results of the FMVM method were highly accurate and consistent with those of the direct visual red blood cell tracking (VRBCT) method.

## 2 Methods and Materials

### 2.1 Setup

**Fig. 1:**
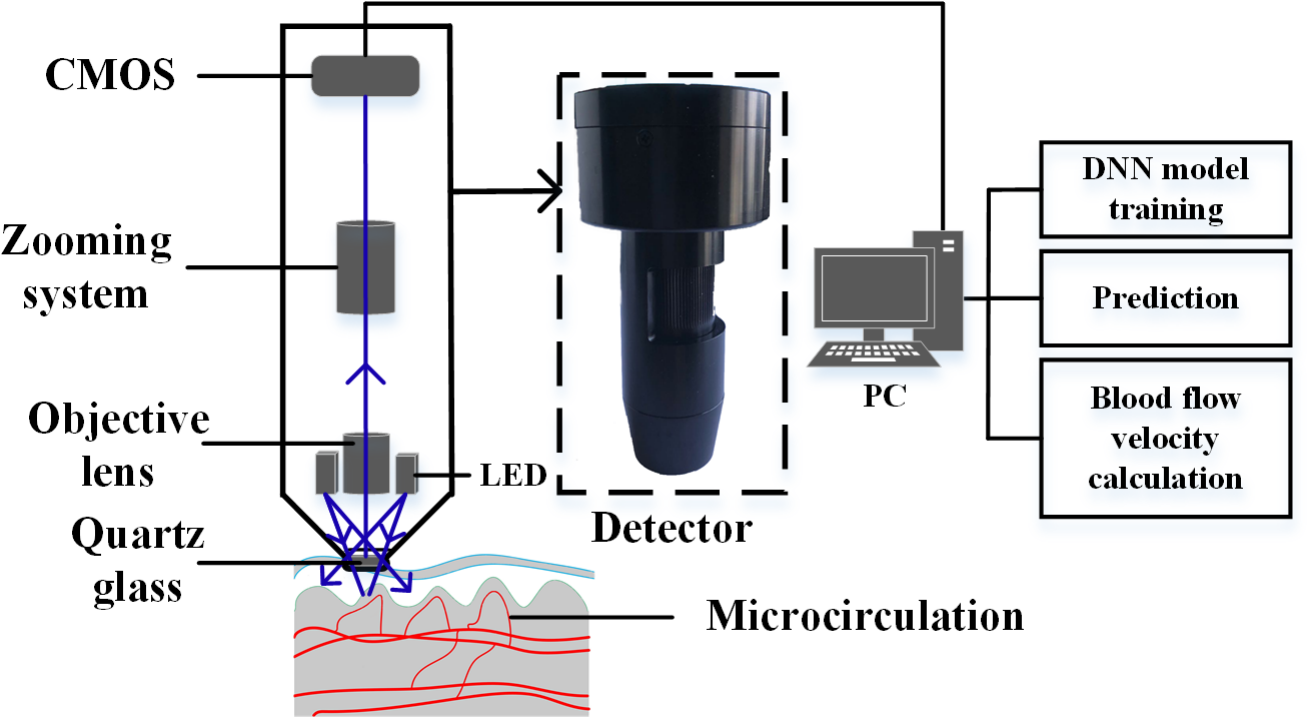
Schematic representation of the experimental device.

The experimental setup is shown in Fig. 1. The system consists of a custom optical detector. The optical system comprises two light-emitting diode sources of wavelength 420 nm and power 60 mW, and an image acquisition system. To obtain high contrast images of microcirculation, an illumination source of wavelength in the strong absorption band of red blood cells (RBCs) must be chosen. In general, the light absorption by RBCs is stronger at the wavelength 420 nm,^28^ which provides a better image contrast than white light. The image acquisition system comprises a complementary metal-oxide semiconductor camera (Aptina, MT9V024) of resolution 744×482  pixels(pixel size of 6×6  μm) and framerate 60 fps, a zooming optical system, and an objective of numerical aperture 0.12. A quartz glass of thickness 1 mm was placed between the objective and the skin. The skin was covered with a material of refractive index similar to that of oil to reduce surface reflection. A personal computer (PC) was used to obtain the optical images and videos. The PC was equipped with a graphic processing unit (NVIDIA 1080Ti) for DNN training and prediction. A custom-made program was used for flow velocity calculations.

### 2.2 Principle of Full-Field Microcirculation Velocity Measurement Method

In the object detection field, Liu et al.^29^ proposed a Single Shot MultiBox Detector (SSD) in 2016, which used a single convolutional neural network to detect the object in an image. In 2017, Chengcheng et al.^30^ proposed a method to improve the SSD algorithm to increase its classification accuracy without affecting its speed. They adopt the inception block to replace the extra layers in SSD and called this method inception SSD. The proposed network can catch more information without increasing the complexity. In this research, SSD_Inception_v2_COCO (Google), which used the network structure of inception SSD, was used for microcirculation identification and segmentation.

Transfer learning is the improvement of learning in a new task through the transfer of knowledge from a related task that has already been learned.^31^ DNNs for complex tasks such as object detection need to be large and deep, resulting in thousands of parameters. This means that training such networks requires huge datasets and computational resources, and the training process might require days or even weeks to complete. Therefore, pretrained model was employed in this research for microcirculation identification and segmentation. The DNN model, SSD_Inception_v2_COCO, was pretrained by COCO dataset (Microsoft) and was used to identify and segment the individual capillaries in this research. We further trained it by microcirculation image dataset in this research.

The microcirculation image dataset was composed of 500 labeled microcirculation images obtained from 12 volunteers. Further, 420 images were used as the training set and 80 images as the test set, for training the DNN model. The image resolution was set to 744×482  pixels. The original images were processed according to the following procedure. First, data augmentation methods, such as flip, random crop and color distortion, and random expansion were utilized in this experiment to expand the dataset. The total number of images in the data training set was increased to 6300. Then, an open source software, LabelImg,^32^ was used for microcirculation image annotation. The capillaries in the microcirculation images were marked by rectangular boxes. A text file containing the capillary location information was generated for each original image using LabelImg. Finally, the original images and the text files of the training set and test set were transformed into two “TFRecords files” for rapid processing using TensorFlow (Google). TensorFlow 1.6 under Python 3.6 was used for model training and forecasting. The training parameters were set as follows: base learning rate—0.01, batch size—10, moment—0.98, weight decay—0.01, and iterations—12,000. The trained DNN model was then used to locate the capillaries. If a microcirculation image is transmitted to the neural network, the location information of all the capillaries can be obtained and marked by rectangular boxes.

**Fig. 2:**
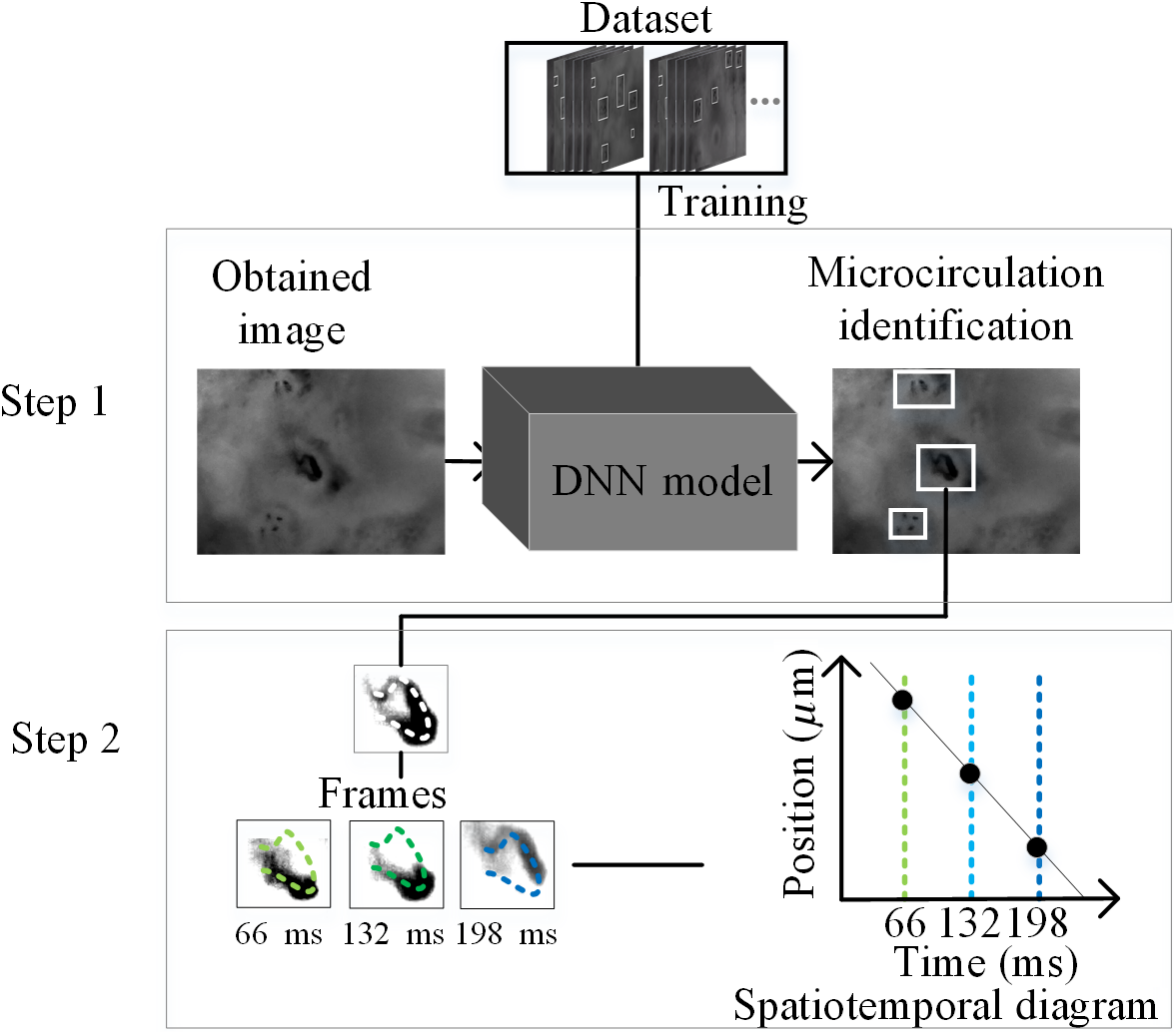
Schematic representation of the FMVM method for blood flow velocity measurement in microcirculation.

Figure 2 shows the schematic representation of FMVM method. The FMVM method involves three steps to measure the full-field blood flow velocities. First, a random frame of video was selected and the DNN model was used to identify and segment the individual capillaries in the frame. Second, the segmented capillaries were processed by threshold segmentation to remove the background, and a skeleton was extracted from the microcirculatory vessel to record the pixel values of the video frames. The skeleton graph extraction method was based on the Khalid, Marek, Mariusz, Marcin algorithm.^33^ The pixel values of the skeleton curve were recorded in one-dimensional (1-D) arrays. The 1-D arrays obtained from the consecutive frames were transformed into a two-dimensional spatiotemporal diagram. Finally, for all capillaries segmented in the first step, step 2 was repeated. The blood flow velocities of all the capillaries were thus obtained simultaneously.

As shown in Fig. 2, the spatiotemporal diagram comprises a straightened skeleton curve with a series of gray-level points. The horizontal and vertical axes indicate the time and spatial axes, respectively. The movement of the RBCs leads to a curvilinear trajectory in the spatiotemporal diagram, and the curvilinear trajectory was connected by a straight line. The slope of the straight line indicates the velocity of blood flow, and the positive and negative values of the slopes indicate the directions of blood flow. By calculating the slope of all the curvilinear trajectories, the average flow velocity can be obtained.

It must be noted that the images in the training and test datasets were not used as samples in the capillary identification and blood flow velocity measurement experiments.

### 2.3 Materials

The main research object of this study is human skin. A large number of capillaries exist in the papillary dermis below the skin surface.^34^ Therefore, the human skin is a window to observe dynamic microcirculation. The microcirculation diagram of the human skin is shown in Fig. 1. To obtain the microcirculation images using an optical imaging system, 12 volunteers were invited. The sampling areas included the arm, face, and other parts the body. Before conducting the microcirculation image and video acquisition experiments, we treated the imaging region by ethanol disinfectant (75%). During the acquisition process, we used a fixed bracket to ensure that the relative position of the optical probe and the sample remained unchanged to avoid any errors in velocity measurement due to jitter. A total of 500 images of different regions were collected for the DNN model training and testing, and 10 videos of dynamic microcirculation were acquired for the blood flow velocity measurement. The volunteers were thoroughly informed of the objectives of this study, and they provided their written consent. Ethics approval was granted by the Third Affiliated Hospital, Sun Yat-sen University, Guangzhou, China.

## 3 Results

### 3.1 Intelligent Identification of Microcirculation

We first evaluated the performance of microcirculation identification by the test dataset. There were 80 images containing 435 capillaries in the test dataset. The probability of the detection rate of capillaries is given as $P_{m\_T}=N_{m\_\mathrm{detect}\_T}/N_{m\_\mathrm{total}}$; the probability of false positive detection rate of capillaries is given as $P_{m\_F}=N_{m\_\mathrm{detect}\_F}/N_{m\_\mathrm{total}}$. In the equation, $N_{m\_\mathrm{total}}$ is the number of total marked capillaries, $N_{m\_\mathrm{detect}\_T}$ is the number of detected capillaries, and $N_{m\_\mathrm{detect}\_F}$ corresponds to the detected mistaken capillaries. The corresponding data are listed in Table 1. From Table 1, the results clearly indicate that the DNN model has high accuracy in detecting microcirculation.

**Table 1:** Performance of the test dataset in microcirculation identification.

$N_{m\_\mathrm{total}}$	$N_{m\_\mathrm{detect}\_T}$	$N_{m\_\mathrm{detect}\_F}$	$P_{m\_T}$	$P_{m\_F}$
435	400	31	92.0%	7.1%

**Fig. 3:**
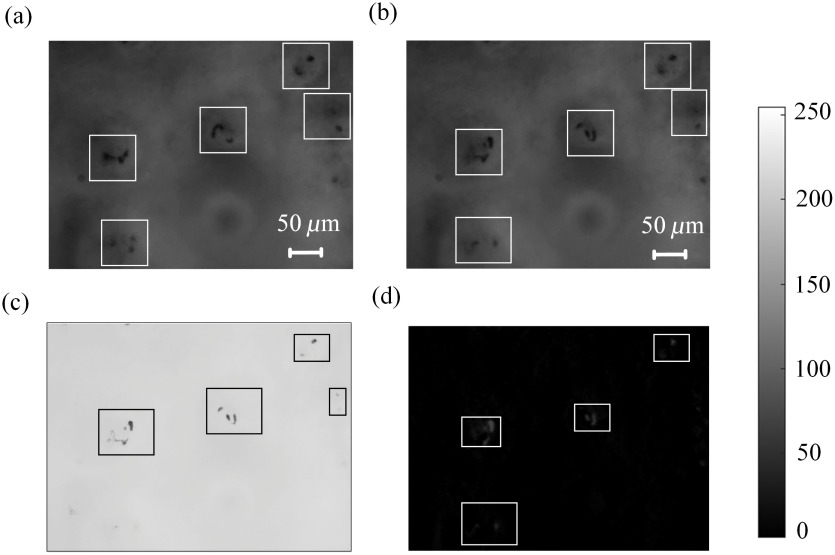
Microcirculation identification results using the DNN model, threshold segmentation method, and frame difference method. (a) and (b) Microcirculation identification by the DNN model; (c) microcirculation identification by threshold segmentation method; and (d) microcirculation identification by frame difference method.

Then, we utilized the trained DNN model to conduct intelligent microcirculation identification. Figures 3(a) and 3(b) show the results of microcirculation identification using the DNN model trained by our dataset. The white rectangles in the figures indicate the boundaries of the capillaries marked by the DNN model. From these figures, it is clear that the DNN model accurately identified almost all the capillaries. Using the DNN model, all the capillaries can be automatically and simultaneously segmented in one video frame.

To evaluate the robustness of the proposed approach, we used the threshold method and frame difference method to separate the individual capillaries from each video frame. Figure 3(c) shows the results of identification of capillaries in Fig. 3(a) by the threshold segmentation method. The results indicate that four capillaries were successfully identified, but one was missed due to the low signal-to-noise ratio of the microcirculation. Figure 3(d) shows the results of identification of capillaries in Figs. 3(a) and 3(b) by the frame difference method. The results of Fig. 3(d) clearly show that four capillaries were identified, but one was missed due to low velocity of blood flow. In the conventional image-processing methods, many complicated situations must be considered for identification and segmentation of a single capillary. Moreover, the result is vulnerable to noise.

The DNN model used in this study requires the training set samples to be highly representative. To obtain more accurate results, maximum number of training samples is required. If the number of training samples is insufficient, achieving an ideal recognition accuracy is difficult. The DNN model can be successfully applied in the identification of capillaries on the skin surface because the characteristics of these capillaries are clear, as shown in Fig. 1, and these capillaries are rarely crossed. Therefore, only 420 images of training dataset were in this method to obtain positive training results.

### 3.2 Blood Flow Velocity Measurement with Spatiotemporal Diagram Method

**Fig. 4:**
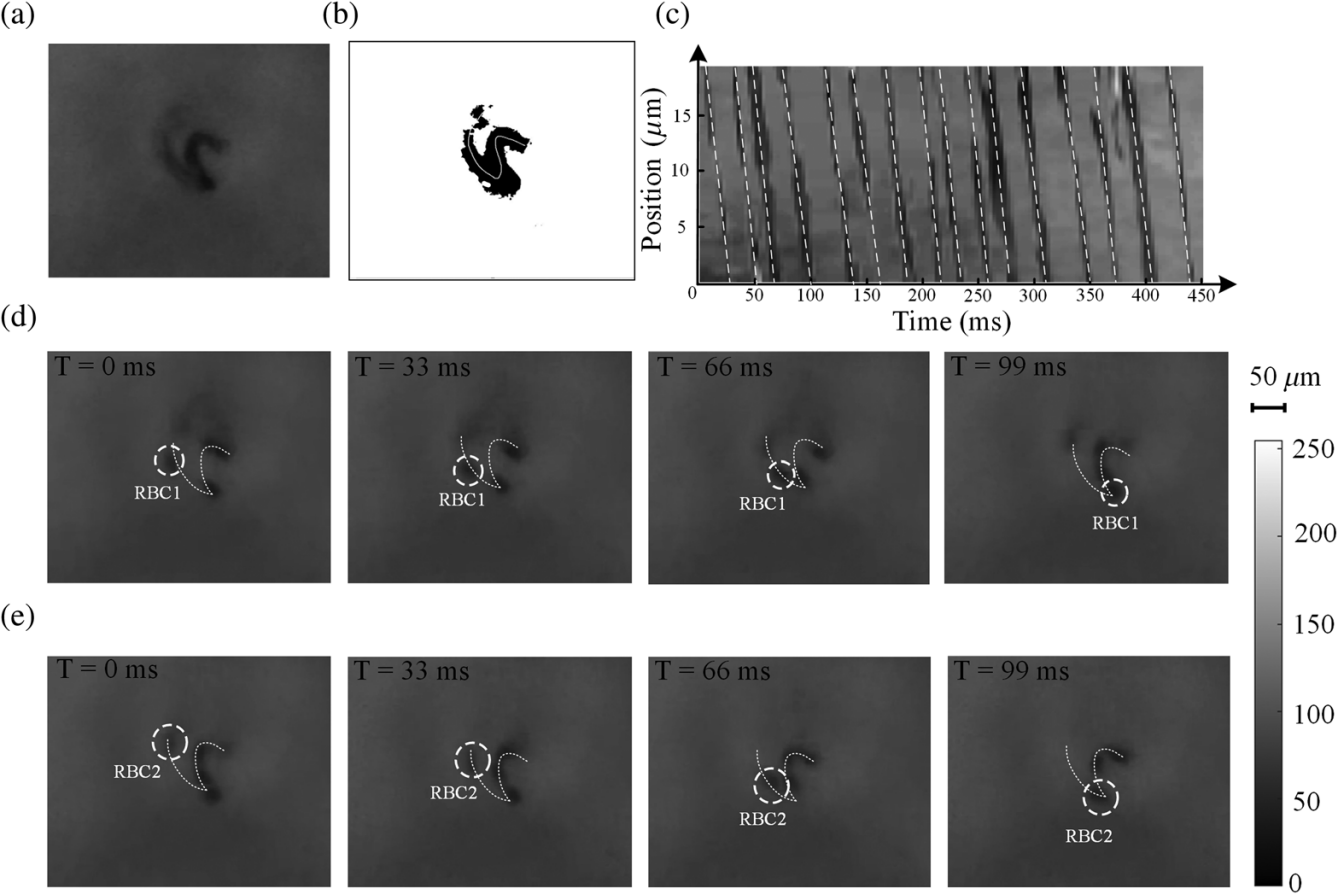
Blood flow velocity measurement experiments using the spatiotemporal diagram and VRBCT methods. (a) A video frame of dynamic microcirculation; (b) capillary and its skeleton (obtained by background removal); (c) spatiotemporal diagram generated using the skeleton curve positions obtained from the video frames; (d) and (e) visual tracking of two RBCs (watch Video 1, 20 MB, MP4 [URL: https://doi.org/10.1117/1.JBO.25.1.016003.1]).

To calculate the blood flow velocity of a single capillary, a video of dynamic microcirculation was obtained from a volunteer. The capillaries were identified and segmented using the DNN model. Figures 4(a)–4(c) show the results of the blood flow velocity measurement experiments using the spatiotemporal diagram method. A video frame of the dynamic microcirculation is shown in Fig. 4(a), which was subjected to background removal as shown in Fig. 4(b). The white curve in Fig. 4(b) indicates the skeleton of the capillary. The pixel values of the curve were acquired from the video frames to generate the spatiotemporal image. Figure 4(c) shows spatiotemporal diagram of the RBCs. Because of the strong absorption characteristic of the RBCs, their trajectory appears dark in the image. The trajectory was connected by a straight line. The slope of the straight line represents the flow velocity of the capillary. The dashed lines in Fig. 4(c) signify the connected straight lines. The average slopes of these lines represent the average flow velocities. From Fig. 4(c), the average flow velocity was calculated as ∼1.2±0.1  mm/s.

We employed the VRBCT method for flow velocity measurement, which is more intuitive,^4^^,^^35^^,^^36^ to evaluate the accuracy of the spatiotemporal diagram method. Using this method, the trajectory of the RBCs can be visualized directly from the video frames. By dividing the path length of the RBCs by time, their flow velocity can be obtained directly. Figures 4(d) and 4(e) show the trajectories of two RBCs. The time interval of the frames was 33 ms. The dynamic microcirculation video has been provided as Video 1. RBC1 was tracked and marked by a dashed circle, as shown in Fig. 4(d)—the velocity of RBC1 was ∼1.0±0.1    mm/s, path length was 98  μm, and the time span of displacement was 99 ms. RBC2 was tracked and marked by a dashed circle, as shown in Fig. 4(e)—the velocity of RBC2 was ∼1.2±0.1  mm/s, path length was 114  μm, and the time span of displacement was 99 ms. The results of this method agree well with those of the spatiotemporal diagram method.

Therefore, both the spatiotemporal diagram and the VRBCT methods can be employed to measure the flow velocity of blood, and their accuracies are comparable. The VRBCT method chiefly depends on mental reasoning, whereas the spatiotemporal diagram method eliminates the need for human intelligence. Therefore, the spatiotemporal diagram method can be effectively used as an automatic blood flow velocity measurement system.

### 3.3 Full-Field Flow Velocity Measurements of Microcirculation with Full-Field Microcirculation Velocity Measurement Method

**Fig. 5:**
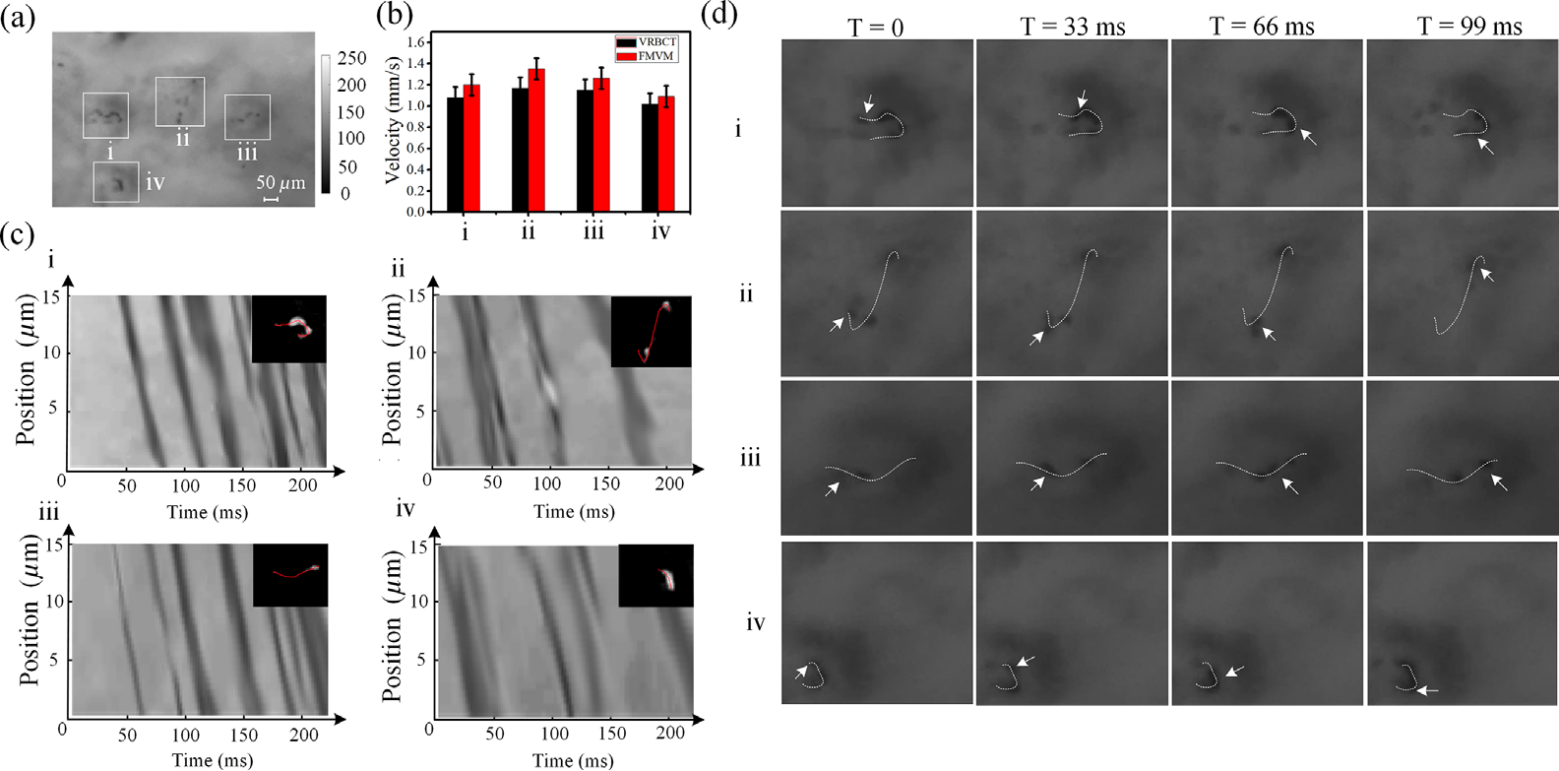
*In vivo* full-field flow velocity measurements of microcirculation. (a) A video frame of microcirculation; (b) flow velocity measurement results of the FMVM and VRBCT methods; (c) spatiotemporal diagrams of capillaries (i)–(iv) [of Fig. 5(a)]; and (d) flow velocity measurements of regions (i)–(iv) [of Fig. 5(a)] using the VRBCT method by tracking the movements of RBCs. Dynamic video of multiple capillaries (watch Video 2, 5 MB, MP4 [URL: https://doi.org/10.1117/1.JBO.25.1.016003.2]).

To verify the feasibility of the *in vivo* full-field flow velocity measurement of microcirculation, an experiment was conducted, and a volunteer was invited to participate in the experiment. We recorded a dynamic microcirculation video of the facial area without injury. Figure 5(a) shows a video frame of the microcirculation. In Fig. 5(a), (i)–(iv) regions indicate the capillaries identified by the DNN model. Each capillary region was segmented. To acquire a microcirculation image with background removal, an individual image was color-reversed (from black to white). As a result, the capillary image of high pixel values was obtained. The skeleton was extracted and marked by a red curve as shown in Fig. 5(c). The spatiotemporal diagrams were generated using the frames of the video, based on the skeleton curves. Figure 5(c) shows the spatiotemporal diagrams of regions (i)–(iv) in Fig. 5(a). The average flow velocities of the capillaries (i)–(iv) were acquired from the dark curvilinear trajectories in the spatiotemporal diagrams as ∼1.2±0.1, ∼1.4±0.1, ∼1.3±0.1, and ∼1.1±0.1  mm/s, respectively.

The VRBCT method was employed for flow velocity measurements in this experiment to verify the accuracy of the FMVM method. The dynamic microcirculation video is provided as Video 2. The RBCs were tracked in the four capillaries. The trajectories of RBCs were marked by dashed curves as shown in Fig. 5(d). Using the path length and time span of the RBCs, the average velocity can be acquired. The average flow velocities of the capillaries calculated by the VRBCT method were ∼1.1±0.1, ∼1.2±0.1, ∼1.2±0.1, and ∼1.0±0.1  mm/s. A comparison of the results of these two methods is given in Fig. 5(b). Considering the results of the VRBCT method as the scale, the average coincidence is 90%.

Using the FMVM method, the blood flow velocity of each capillary can be acquired. The statistics, such as the mean and variance of the velocity, can be acquired, which are valuable in clinical assessments of conditions. Because the capillaries are automatically identified by the DNN model, a fully automatic flow velocity measurement can be achieved by combining the DNN model and the spatiotemporal diagram analysis method.

## 4 Discussion and Conclusion

In this research, we developed an *in-vivo* optical microscope with a 420-nm light source for microcirculation imaging and further proposed a blood flow velocity measurement method based on intelligent object identification. By employing the DNN model, the method achieves high accuracy of microcirculation identification and flow velocity measurement. The DNN model was trained using a dataset of microcirculation images obtained from humans, in this study. The results obtained by employing the DNN model in the microcirculation identification experiment presented higher accuracy of microcirculation identification and segmentation than that by the threshold method and frame difference method. Further, a flow velocity measurement experiment was conducted using the spatiotemporal diagram method, and the results were consistent with those of the direct VRBCT method. Finally, an *in-vivo* full-field microcirculation velocity experiment was conducted, in which the capillaries were segmented from the whole image using the DNN model. In addition, the blood flow velocities of the capillaries were calculated using the spatiotemporal diagram method and the results were compared with those of the direct VRBCT method. The results showed good agreement with those of the VRBCT method, thereby demonstrating that the proposed FMVM method can be effectively employed for FMVM. Furthermore, using this method, a few statistics of the flow velocities in the full-field imaging region, such as mean and variance, can be acquired, which are valuable for clinical assessment of certain conditions.

In this research, we use deep learning-based vascular recognition to segment microvessels and use spatial-temporal diagram method to calculate blood flow velocity. Compared with the traditional machine learning method, the deep learning method in this work can intelligently identify the microvessels and directly obtain the number and location information of microvessels in the field of view, which is very helpful for full-field blood flow velocity measurement. In the case of small sample size, using a traditional machine learning method will get better results. In the case of large sample size, using a deep learning method can get better results. Our method based on deep learning has better adaptability to complex scenes.^20^

Because the dataset used in this work is small (only 12 volunteers participated in the experiment), the effect of DNN model on microcirculation identification will suffer from overfitting. Therefore, data augmentation was used to increase the training dataset in this work. To obtain better adaptability, a larger and wider dataset is needed to train the DNN model. In particular, the DNN model used in this research was trained only using capillaries that were free of overlap, and it is not suitable for the condition of overlapped capillaries. In this situation, a larger dataset containing overlapped capillaries is needed to train the DNN model.

In theory, the upper and lower limits of the FMVM are determined by the frame rate of the video acquired. A frame rate of 60 fps, length of an observable single microvessel of ∼400  μm, and optical limit resolution of 0.18  μm (waveform of 420 nm) were used in this study, for which the upper and lower limits of the flow velocity were obtained as 23.95 and 0.01 mm/s, respectively. The upper and lower limits of the speed measurement system vary based on image sensors of different frame rates. According to a previous report, the flow velocity range of microcirculation is 0.1 to 2.2 mm/s.^37^ Therefore, the FMVM method is effective for blood flow velocity measurement of microcirculation in humans.

The FMVM method performs well when a certain period of time exists between adjacent RBCs, such that the diagonal streaks in the spatiotemporal diagram are well segmented. This research did not consider multiple RBCs flowing close to each other, such as sickle cell anemia case, in its experiments. However, if multiple RBCs flow very closely so that they cannot be distinguished by the microscope used in this research, they will be recognized as one large RBC and a wide diagonal will be created in the spatiotemporal diagram, where the slope of diagonal will still represent the flow velocity of microcirculation. If the RBCs accumulate and the diameter after aggregation exceeds the field of view of the microscope, the light and dark diagonal streaks in the spatiotemporal diagram cannot be produced and the FMVM method will fail.

In this research, we manually selected the diagonal streaks in the spatiotemporal diagram and calculated their slopes. If a large flow of RBCs occurs during the recording time frame, the time required to calculate the velocity of each RBC manually will increase greatly, rendering the process very cumbersome. This is one of the few limitations of this technology in clinical applications. However, realizing an automatic straight-line fitting and slope calculation of the diagonal streaks in the spatiotemporal diagram are theoretically feasible using image processing, which will be the objective of our future work.

The FMVM method based on intelligent object identification can potentially avoid the miscalculation of microcirculation and provide accurate flow velocity data. This method has been proved to be applicable to microcirculation of human skin, including face, arm, and other parts. Moreover, this method is simple and cost effective. Thus, the proposed FMVM method holds potential for clinical applications, especially in the diagnosis of diabetes and cancer, and for meticulous observation.
